# Supplementary material for: Salivary Cytokines as Biomarkers for Oral Squamous Cell Carcinoma: A Systematic Review
Source: Int J Mol Sci. 2021 Jun 24;22(13):6795. doi: 10.3390/ijms22136795 (PMC8267678; doi:10.3390/ijms22136795)
Supplement: Supplementary file 1 [file ijms-22-06795-s001.zip › ijms-1264478-supplementary.pdf]

International Journal of
Molecular Sciences

MDPI

Review

Salivary Cytokines as Biomarkers for Oral Squamous Cell Carcinoma: A Systematic Review

Elena Ferrari [1], Margherita E. Pezzi [2], Diana Cassi [3], Thelma A. Pertinhez [1,*], Alberto Spisni [1] and Marco Meleti [1,2]

1 Department of Medicine and Surgery, University of Parma, Parma, Italy
2 Centro Universitario Odontoiatria, University of Parma, Parma, Italy
3 Dentistry and Oral and Maxillofacial Surgery, Department of Surgical, Medical, Dental and Morphological Science with interest in Transplant Oncological and Regenerative Medicine, University of Modena and Reggio Emilia, Modena, Italy

* Correspondence: thelma.pertinhez@unipr.it

SUPPLEMENTARY TABLES

Table S1. Risk of specific bias for the included studies.

Ref.	Q1	Q2	Q3	Q4	Q5	Q6	Q7	Q8	yes (%)	Risk of bias
[27]	✓	✓	✓	✓	✓	✓	✓	✓	100	no
[28]	✓	✓	✓	✓	✓	✓	✓	✓	100	no
[29]	✓	✓	✓	✓	✓	✓	✓	✓	100	no
[32]	✓	✓	✓	✓	–	–	✓	✓	75	+
[33]	✓	✓	✓	✓	✓	✓	✓	✓	75	+
[34]	✓	✓	✓	✓	–	–	✓	✓	75	+
[35]	✓	✓	✓	✓	✓	✓	✓	✓	100	no
[36]	✓	✓	✓	✓	✓	✓	✓	✓	75	+
[37]-	✓	✓	✓	✓	–	–	✓	✓	100	no
[38]	✓	✓	✓	✓	✓	✓	✓	✓	100	no
[39]	✓	✓	✓	✓	✓	✓	✓	✓	75	+

[40]	✓	✓	✓	✓	✓	✓	✓	✓	100	no
[41]	✓	✓	✓	✓	✓	✓	✓	✓	100	no
[42]	✓	✓	✓	✓	✓	✓	✓	✓	100	no
[43]	✓	✓	✓	✓	✓	✓	✓	✓	100	no
[44]	✓	✓	✓	✓	✓	✓	✓	✓	100	no
[45]	✓	✓	✓	✓	✓	✓	✓	✓	100	no
[46]	✓	✓	✓	✓	✓	✓	✓	✓	100	no
[47]	✓	✓	✓	✓	✓	✓	✓	✓	100	no
[48]	✓	✓	✓	✓	✓	✓	✓	✓	100	no
[49]	✓	✓	✓	✓	✓	✓	✓	✓	100	no
[50]	✓	✓	✓	✓	✓	✓	✓	✓	100	no
[51]	✓	✓	✓	✓	–	–	✓	✓	75	+
[52]	✓	✓	✓	✓	✓	✓	✓	✓	100	no
[53]	✓	✓	✓	✓	✓	✓	✓	✓	100	no
[54]	✓	✓	✓	✓	✓	✓	✓	✓	100	no
[55]	✓	✓	✓	✓	✓	✓	✓	✓	100	no

Questions: "Q1. Were the criteria for inclusion in the sample clearly defined? Q2. Were the study subjects and the setting described in detail? Q3. Was the exposure (quantitative analysis of salivary cytokine) measured in a valid and reliable way? Q4. Were objective, standard criteria used for measurement of the condition? Q5. Were confounding factors identified? Q6. Were strategies to deal with confounding factors stated? Q7. Were the outcomes (OSCC disease) measured in a valid and reliable way? Q8. Was appropriate statistical analysis used?

Answer: √: yes; –: no.

Risk of bias: *no*: no risk; +: low risk (25% of the answers are negative).

Negative points in Table S1 are in response to Question number 5 and 6, regarding confounding factors. In the reviewed studies, potential confounders were chronic or acute inflammatory conditions (periodontitis, gingivitis), systemic diseases, and unhealthy habits (such as alcohol abuse, tobacco). In most of the articles, confounding was reduced by restricting the study population; for example, subjects with comorbidities were not included in the study population. Alternatively, a matching strategy was used; for example, selected cases were matched to controls by means of their comparable periodontal status. When confounders were not assessed, prevented, or excluded we assigned a negative point.

International Journal of *Molecular Sciences*

MDPI

Table S2 - Demographic profile of OSCC patients involved in the included studies.

	Ref.	cytokine	Number of patients	Country of origin	Gender		Mean age ± SD (y)
					F	M	
1	[27]	IL-1α, IL-6, IL-8, TNF-α, HCC-1, MCP-1, PF-4	66	Spain	33	33	65 ± 15.6
2	[28]	IL-10, IL-13, IL-1RA, IL-4	30	Pakistan	6	24	50.3 ± 15.6
3	[29]	IL-1α, IL-6, TNF-α	28	Croatia	6	22	61.9
4	[32]	IL-6, IL-8	35	Pakistan	11	24	46.7 ± 14.4
5	[33]	IL-1α, IL-6, IL-8, osteopontin	19	Japan	10	9	60.9
6	[34]	IL-1, IL-6, IL-8, TNF-α	13	USA	3	10	59.5 ± 6.7
7	[35]	IL-6, TNF-α	19	Croatia	7	12	54.2 ± 8.4
8	[36]	IL-1α, IL-6, IL-8 TNF-α, VEGF-a	18	USA	6	12	56.5 ± 13.6
9	[37]	IL-1α, IL-6, IL-8, GM-CSF	30	Iraq	14	16	53
10	[38]	IL-1α, IL-6, IL-8, TNF-α	9	Iran	5	4	71.3 ± 7.8
11	[39]	IL-10 ,TNF-α, TGF-α, VEGF	78	Poland	7	71	63.8
12	[40]	IL-1α, IL-8	60	USA	3	57	51.7
13	[41]	IL-8	25	India	9	16	53.2 ± 11.4
14	[42]	IL-6, IL-8, IL-1β, TNF-α, IFN-γ, MIP-1β, Eotaxin, GRO	41	Taiwan	5	36	55.0
15	[43]	IL-6	100	India	32	68	21-90*
16	[44]	IL-8	100	India	32	68	21-90*
17	[45]	TNF-α	30	India	9	21	57.8 ± 9.4
18	[46]	TNF-α	100	India	32	68	21-90*
19	[47]	TNF-α	30	India	4	26	24-74*
20	[48]	IL-6, IL-8	18	USA	7	11	59.4
21	[49]	IL-8, IL-1β	35	Serbia	5	30	60.9 ± 12.3
22	[50]	IL-6	25	India	4	21	49
23	[51]	IL-17	25	Iraq	8	17	57.9 ± 10.4
24	[52]	IL-8, IL-6, VEGF, MIP-1β, IP-10, IL-1β, INF-γ, IL-5	20	Italy	10	10	64.9 ± 14.2
25	[53]	IL-1β	16	Japan	6	10	56.3 ± 5.6
26	[54]	IL-6	27	Japan	11	16	70.7 ± 8.6
27	[55]	IL-6	27	Japan	11	16	70.7 ± 8.6

* range
